# Diagnosing orthopaedic infection by identifying neutrophils in whole histology slide images with machine learning trained on publicly available datasets

**DOI:** 10.1302/2046-3758.153.BJR-2024-0587.R2

**Published:** 2026-03-11

**Authors:** Kieran Bentick, Hollie Wilkinson, Ali Al Khader, Helen McCarthy, Karina Wright, Theocharis Kyriacou, Adrienne M. Flanagan, Paul Cool

**Affiliations:** 1 Robert Jones and Agnes Hunt Orthopaedic Hospital NHS Foundation Trust, Oswestry, UK; 2 Keele University, Keele, UK; 3 Department of Microbiology, Pathology and Forensic Medicine, The Hashemite University, Zarqa, Jordan; 4 Royal National Orthopaedic Hospital NHS Foundation Trust, Stanmore, UK; 5 University College London Cancer Institute, London, UK; 6 Computer Science, York St John University, York, UK

**Keywords:** Orthopaedic infection, Prosthetic joint infection, Machine learning, Neutrophils, Artificial intelligence, YOLO (You Only Look Once), infection, Periprosthetic joint infection (PJI), blood, revision surgery, periprosthetic infection, prosthetic joint, orthopaedic infection, revision joint arthroplasty, blood cells, McNemar test

## Abstract

**Aims:**

This study examines the ability of YOLO (You Only Look Once) 11x, a widely used and state of the art object detection model, trained on publicly available datasets, to identify and count neutrophils in tissue samples taken at prosthetic joint revision surgery, with the objective of automating a laborious but necessary part of the diagnostic workup for periprosthetic joint infection.

**Methods:**

Three datasets containing blood film microscopic slides with neutrophils were downloaded, combined, and labelled. The resulting dataset of 3,923 images was augmented with ten additional histological slides from periprosthetic tissue, taken at the time of revision surgery (5 infected, 5 sterile), and split into training (70%), validation (20%), and test (10%) sets. The dataset was used to train YOLO 11x object detection model optimized for a mean average precision above 50%. The trained network was tested on a ground truth specimen and histological whole slide images from 19 additional cases, previously unseen by the model, for validation. The threshold for diagnosis of infection on histological sections was set at more than five neutrophils per 0.2 mm^2^ (equivalent to one high-powered microscope field).

**Results:**

The model performed well as ground truth image returned precision at 82%, recall (sensitivity) 79%, and F1 harmonic mean 80%. When assessed against formal histopathological, microbiological, and multidisciplinary team (MDT) diagnosis, precision was 78%, 80%, and 90%; recall 78%, 89%, and 82%; and F1 score 78%, 84%, and 86%, respectively. Against the definitive MDT diagnosis, our model identified nine out of the ten infected cases and excluded seven out of nine cases that were not infected.

**Conclusion:**

This study demonstrates ability of the trained model to identify neutrophils in tissue taken at revision surgery and could assist in diagnosis of periprosthetic infection. Further work is needed to improve confidence in the identifications and diagnostic accuracy of periprosthetic infection.

Cite this article: *Bone Joint Res* 2026;15(3):238–247.

## Article focus

- This study evaluates the ability of YOLO (You Only Look Once) 11x object detection model, trained on publicly available datasets, to identify and count neutrophils in whole histology slides, and to provide information from which a diagnosis of periprosthetic joint infection (PJI) can be derived.

## Key messages

- YOLO 11x can be trained to identify neutrophils in whole slide histological specimens and deliver a value for the number of neutrophils per high-powered field across the whole sample.
- This can reliably diagnose PJI with confidence comparable to current diagnostic approaches.

## Strengths and limitations

- This model examines the whole slide, rather than a sample of high-powered fields, ensuring areas are not overlooked.
- The model delivers an observable output identifying and marking each neutrophil, as well as providing a numerical value of neutrophils per high-powered field, thereby demonstrating the basis upon which a diagnosis of infection can be made.
- This is a retrospective study using stored histological specimens, a limitation of which is the possibility of selection bias. Further prospective work using different laboratories, tissue processing techniques, and scanners using larger cohorts could improve confidence and generalizability.

## Introduction

Periprosthetic joint infection (PJI) is a major complication following joint arthroplasty surgery, affecting 1% to 2% of all joint arthroplasties in the UK.^1^ The management options and outcome of PJI are influenced by an accurate and timely diagnosis,^2^ and novel diagnostic approaches are being explored.^3-7^

Diagnostic methods have been debated, but there is no gold-standard test and no universally accepted definition for diagnosing infection. Nevertheless, the Musculoskeletal Infection Society (MSIS) international consensus meetings in 2011 and 2018 set major and minor criteria for reaching a diagnosis.^8-10^ Histological identification of neutrophils in periprosthetic tissue is a highly weighted minor criterion. However, it is a time-consuming task performed by pathologists, who are in short supply.

The threshold for histopathological diagnosis has been a subject of debate.^8-12^ However, a neutrophil count of five or more per high-powered field has optimum sensitivity and specificity.^10^ Neutrophils within blood vessels are excluded from the count, as this is a normal finding and has no relationship with infection.^10-14^ Positive microbiological cultures also support a robust diagnosis of periprosthetic infection,^15^ although without histological confirmation this may represent a false positive result.

**Fig. 1:**
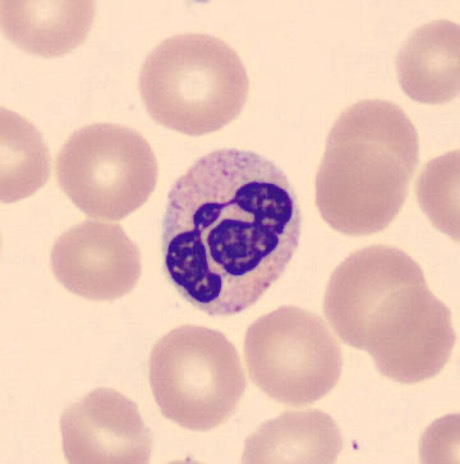
A neutrophil seen in a blood film.^17^ Visible is a dark lobulated nucleus within a lighter stained cytoplasm set among anucleated red blood cells.

**Fig. 2:**
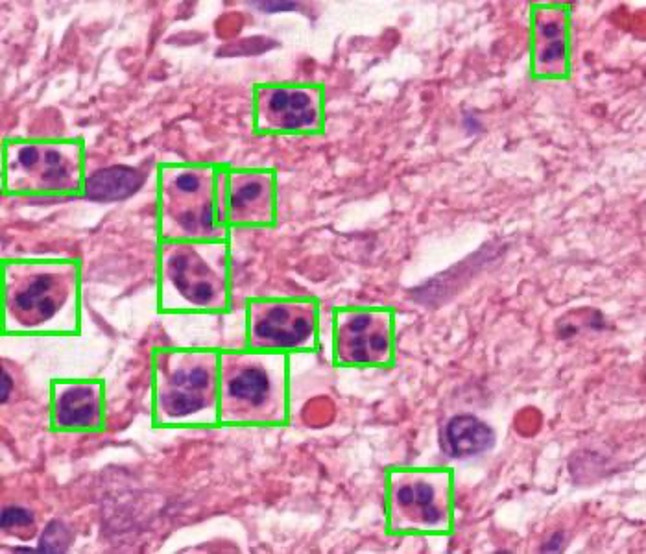
Haematoxylin and eosin-stained tissue sample of a patient with a periprosthetic joint infection supported by the presence of several neutrophils (green boxes).

Neutrophils have a characteristic histological appearance and are usually recognizable in haematoxylin and eosin-stained (H&E) formalin-fixed paraffin-embedded (FFPE) tissue sections (Figure 1 and Figure 2).^16^ However, identification is not always straightforward. Enzyme histochemistry with Naphthol AS-D-chloroacetate esterase can help,^17-19^ but is rarely used in the UK as it is considered inefficient and does not significantly enhance the diagnostic process.

The number of neutrophils in a histological slide can vary within different parts of the section. It is recommended to assess at least five to ten high-powered fields within the section.^11,12,20^ Furthermore, neutrophil counts can have high inter- and intraobserver variability.^13,14,21-27^ Finally, not all centres undertaking revision arthroplasty have on-site pathologists or laboratories in the diagnostic pathway.

To support pathologists to provide a more accurate and efficient diagnostic service, it would be advantageous to utilize machine-learning techniques to identify neutrophils. Machine-learning methods have already been shown to be useful in histopathology, and are effective in identifying different types of white blood cells in peripheral blood.^28-40^ Machine-learning models such as YOLO^37^ and R-CNN^38-40^ models have performed well in identifying cell types accurately. However, this task is more challenging in solid tissue because of the greater spectrum of histological features therein.

Computer assessment of images is used within radiology and histopathology with a range of approaches and models used to differentiate abnormal tissues, cells, and areas within radiological images. Many studies have tested image classification models^32,34-36,39^ and object detection models.^33,37,38,40^ Image classification models determine what class the whole image falls within. This produces an outcome interpreted by the algorithm which can classify an image into a particular group. Object detection models determine whether an instance of an object is present within an image, and provide the location of the object visually represented with a bounding box around it, as well as an output with coordinates and size. In this way, quantum can be calculated and interpreted by an observer. Image segmentation goes a step further and ascertains the precise boundary of the object, providing an area within the image taken up by the object.

Training the model can pose challenges, with vast quantities of annotated images and graphics processing unit (GPU) time required.^41^ The GPU memory and time varies from model to model, with some, such as Faster R-CNN, being more resource-heavy and YOLO-based models being a little lighter. Advances have been made in self-supervised learning methods, reducing the requirement for large volumes of labelled images.^42^ This can deliver a model with excellent ability to classify an image, however the precise features upon which the model bases the outcome remain unknown and may not be generalizable.

The accuracy and speed of the models vary, with models such as Faster R-CNN being marginally more accurate but slower than can be used for real-time processing, and YOLO-based models being markedly faster, enough for real-time processing in a video image without any perceptible lag.^43^

Approaches to object detection also differ by model, with CNN-based models using a sliding window approach where the classifier is sequentially run at intervals over portions of the image. R-CNN generates object proposal boxes and the classifier is executed sequentially on each. YOLO uses a unified neural network architecture concurrently undertaking classification tasks and predicting bounding boxes, using stacked convolutional and connected layers. It delivers prediction of the central point, height, and width (x, y, h, w) for each object detected, represented on the image as the bounding box. This provides the benefit of the model assessing the image as a whole, and effectively providing context and improving generalizability.^43^

ResNet, CNN, and SVM have been demonstrated to provide effective image classification when trained on either supervised or self-supervised parameters. PAIGE prostate is a system based on CNN that is being trialled within a number of hospitals in the NHS in the UK.^30,32,34-36,44,45^

ResNet 50 with single shot detector (SSD), Faster R-CNN, and YOLO-based models trained in a supervised manner have been used in counting breast cancer cells and providing cell counts in a peripheral blood film.^33,37,38,40^

Faster R-CNN has proved competent in segmentation of cross-sectional brain images to segment tumours.^29^

Three studies have evaluated machine-learning methods for use in the diagnosis of PJI.^46-48^ Chen et al^46^ employed conventional machine-learning algorithms to evaluate clinical, biochemical, microbiological, and histopathological parameters taken in combination to stratify risk of infection building in a clinically applicable model. This differs considerably from the methods we are proposing.

Tao et al^47,48^ have provided two studies evaluating ResNet 50, EfficientNet V 2-S, and CAMEL2 in both supervised and self-supervised trained models to classify whole slide images of samples removed at the time of prosthetic joint revision into infected and non-infected cases. They used a range of CNN-based systems and a hybrid convolutional network/centroid aware metric learning network. These studies have demonstrated the ability of models to effectively identify infection within their population set, although to date none have been introduced into clinical practice. However, the lack of defined criteria in the diagnostic features obscures the basis for conclusions and makes generalization of the model difficult.

For this project YOLO 11x was selected. This is a state-of-the-art object detection method, with YOLO demonstrating accuracy in identification of blood cells in peripheral blood films,^37^ while being efficient and having the ability to return identification in real time. It is highly accurate and adaptable, making it an ideal choice for this project.

This paper explores the possibility of using a computer model trained on publicly available datasets with supervised methods to count neutrophils in whole slide histology images to aid the diagnosis of PJI. Providing a cellular count with feedback ensures that the pathologist is kept in the loop in the diagnostic process.

## Methods

Use of specimens was in line with guidance from the Human Tissue Authority and Human Tissue Act 2004.^49^ The methods were carried out in accordance with the Declaration of Helsinki.^50^

Three publicly available datasets were used for training the model: the “Blood Cell Images” dataset;^51^ the dataset used by Acevedo et al^52^ in training their CNN; and the “Raabin-WCC Data” dataset.^53^

These publicly available datasets were merged, giving a total dataset of 3,923 images, all at 100× magnification with resolution between 320 × 240 and 640 × 480, and varying image quality. Most images contained a single neutrophil in a background of red blood cells. However, some images contained two or three neutrophils. All images were manually annotated with a corresponding label file in normalized YOLO format using LabelImg software.^54^ Images were scaled down to 80 × 80 times resolution to produce a neutrophil within the image of approximately 40 × 40 pixels, corresponding to a comparative size of neutrophil within scanned histology haematoxylin and eosin-stained sections. No additional steps or standardization of samples were undertaken in preparation of the slides, and no additional augmentation was undertaken in preparation of the images for training or testing.

The combined dataset was randomly split into a training set of 2,746 images (70%), validation set of 784 images (20%), and test set of 393 images (10%). First training was done with Ultralytics YOLO 11x using a batch size of 256, image size 80, and default image augmentation, within the algorithm, including mosaic augmentation over 100 epochs. Evaluation resulted in 99% accuracy and 1% false positive rate on the test set of blood images.

A second set of scanned histology images was randomly selected from ten patients who had revision joint arthroplasty surgery (five with infection and five without) to augment training. The set contained 640 image patches at 224 × 224 pixels. Neutrophils were approximately 40 × 40 pixels in size. All image patches were independently annotated by two of the authors (PC, KB). Discrepancies were reviewed, and where agreement was not reached the image was discussed with a musculoskeletal pathologist (AMF) to agree on a ground truth set. A second round of training was performed using this second set of histological images (training parameters in Supplementary Material).

**Fig. 3:**
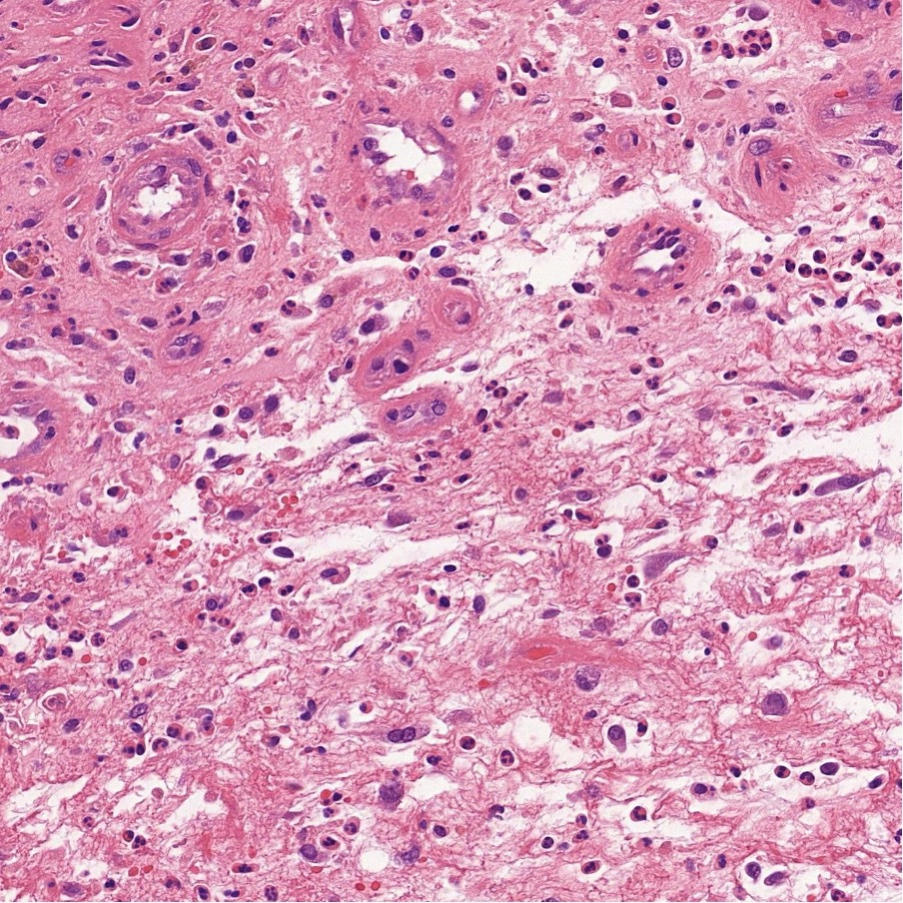
The image patch.

The weights of the second training were used to test the identifications on an image patch (Figure 3), and against a test set of 19 patients who had periprosthetic tissue samples taken for histological evaluation as part of investigation for possible PJI. This included ten patients with infection and nine patients without infection, whose formalin-fixed and paraffin-embedded histology slides were selected at random from the archive. The diagnosis of infection was based upon the ultimate conclusion the infection multidisciplinary team (MDT) reached following evaluation of the clinical picture, microbiological and histopathological results, and in line with MSIS criteria.

The slides were scanned on a 3DHistech single slide scanner (3DHistech, Hungary). The scanner was calibrated with a Heayzoki 0.01 mm microscope calibration slide. Calibration confirmed that the scanner had a resolution of 4,050 pixels per mm. The whole slide images (Mirax, mrxs format) were tiled into images representing one high-powered field (0.2 mm^2^) using Openslide Python 1.3.0 software. This was selected to replicate closely the 0.196 mm^2^ provided by conventional light microscope.^55^ All slides had been reported by expert musculoskeletal histopathologists (AMF, AAK), and in all cases culture results were available.

The agreed joint identifications of neutrophils by PC and KB were subsequently reviewed by two expert pathologists (AMF, AAK), who agreed on identifications, but removed four due to uncertainty. Any neutrophil about which there was any doubt was considered negative, as we hoped to achieve a model where we could be confident that any object identified as being a neutrophil was true, thereby minimizing false positives. This resulted in an agreed ground truth file that was used for testing.

The image patch was annotated and neutrophils identified by two observers (PC, KB), subsequently agreed at a consensus meeting. The precision, recall, and F1 harmonic mean was calculated between the two authors and with the consensus (Table I).

**Table I.** The precision, recall, and F1 harmonic mean was calculated between authors and the joint identification assessing interobserver variation.

Author	Precision	Recall	F1
PC and KB	0.75	0.68	0.71
Joint and PC	0.86	0.98	0.92
Joint and KB	0.90	0.85	0.88

**Fig. 4:**
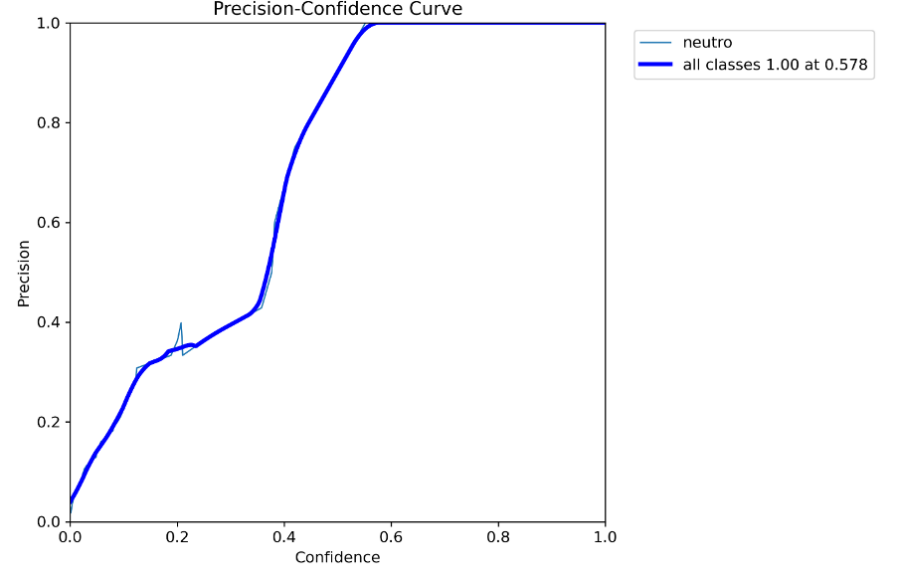
Confidence-precision curve forming the rationale of selection of 50% confidence upon which to base the classifier.

Neutrophils were detected in the image patch with YOLO 11x and the inference slicer (Roboflow, USA) to facilitate identification of small objects using a 512 sliding window with 30% overlap. Boundary boxes of the identifications were compared with the ground truth by calculating the intersection over union (IOU). If the IOU of the identification was greater than 50%, an identification was true positive, otherwise the identification was false negative (Figure 4). All other identifications by the algorithm were false positives.

### Statistical analysis

Statistical analysis was undertaken using R Statistics (R Foundation for Statistical Computing, Austria). The McNemar test (R statistics exact2 × 2 package) was used to compare model performance, and a p-value < 0.05 was considered significant.

## Results

The computer model was tested on the ground truth image patch in Figure 3, and the results are shown in Table II.

**Table II.** F1 is the harmonic mean between precision (positive predictive value) and recall (sensitivity).

Patch	Count	Parameter	Value
True positives	101	Precision	0.82
False positives	22	Recall	0.79
False negatives	27	F1 score	0.80

**Fig. 5:**
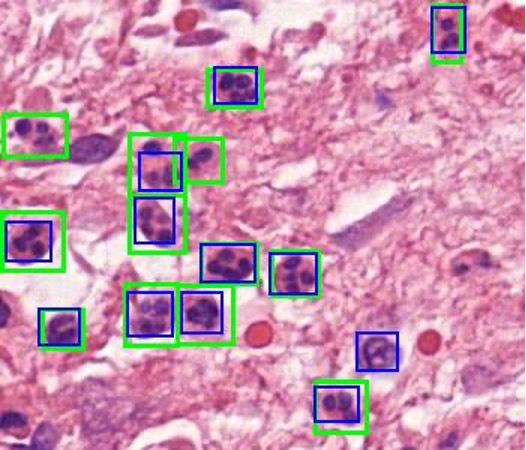
Image of identifications by the model (blue boxes) and ground truth labels (green boxes) on the Image patch. There are two false negatives (green box only), one false positive (blue box only), and 11 true positives (green and blue box).

An example of identifications in comparison to the ground truth (Figure 2) is shown in Figure 5.

Further testing was performed on samples from 19 patients with or without infection who had undergone prosthetic joint revision. Whole slide images were scanned and divided into patches equivalent to one high-powered field as described (Table III). Neutrophils were identified on all these patches (13,786).

**Table III.** Results of classifications comparing histology, microbiological cultures, and multidisciplinary team (MDT) assessment with the computer identifications.

Case	Patches	Median (IQR)	Histology	Microbiology	MDT	Computer
Case 1	339	167 (45 to 263)	Infected	Growth	Infected	Infected
Case 2	165	27 (1 to 18)	Infected	Growth	Infected	Infected
Case 3	1,579	20 (3 to 14)	Infected	Growth	Infected	Infected
Case 4	3,625	11 (2 to 9)	Infected	Growth	Infected	Infected
Case 5	3,376	9 (2 to 8)	Infected	Growth	Infected	Infected
Case 6	96	7 (1 to 8)	Infected	No growth	Infected	Infected
Case 7	832	7 (2 to 8)	Uncertain	Growth	Infected	Infected
Case 8	34	6 (3 to 8)	Not infected	Growth	Infected	Infected
Case 9	431	5 (1 to 6)	Not infected	No growth	Not infected	Infected
Case 10	504	5 (2 to 6)	Infected	Growth	Infected	Infected
Case 11	385	3 (1 to 3)	Not infected	No growth	Not infected	Not infected
Case 12	505	3 (1 to 3)	Not infected	No growth	Not infected	Not infected
Case 13	397	3 (1 to 3)	Infected	No growth	Infected	Not infected
Case 14	239	3 (1 to 4)	Not infected	No growth	Not infected	Not infected
Case 15	321	3 (1 to 3)	Uncertain	No growth	Not infected	Not infected
Case 16	673	2 (1 to 2)	Infected	Growth	Infected	Not infected
Case 17	46	2 (1 to 2)	Not infected	No growth	Not infected	Not infected
Case 18	218	2 (1 to 2)	Not infected	No growth	Not infected	Not infected
Case 19	21	1 (1 to 1)	Not infected	No growth	Not infected	Not infected

A specimen was classified by the computer model as ‘infected’ or ‘not infected’ if the median neutrophil count was greater than five per high-powered field. However, a management decision to treat the patient as having a periprosthetic infection was made by combining results of microbiological culture, histological findings, and the clinical assessment following discussion at a MDT meeting.

Each case is detailed in Table III, and confusion tables, precision, recall, and F1 scores comparing the computer classification with the outcomes (histology, microbiology, and MDT diagnosis) are shown in Table IV.

**Table IV.** Truth tables, precision, recall, and F1 harmonic means for comparison of the model with histology result, microbiology result, and multidisciplinary team (MDT) outcome.

Histology					
Computer	Infected	Not infected	Uncertain	Precision (PPV)	78%
Infected	7	2	1	Recall (Sensitivity)	78%
Not infected	2	6	1	F1 score	78%
**Microbiology**					
Computer	Growth	No growth		Precision (PPV)	80%
Infected	8	2*		Recall (Sensitivity)	89%
Not infected	1	8		F1 score	84%
**MDT diagnosis**					
Computer	Infected	Not infected		Precision (PPV)	90%
Infected	9	1		Recall (Sensitivity)	82%
Not infected	2	7		F1 score	86%

* One case with proven infection received antibiotic treatment prior to sample being taken and returned no growth.

The model incorrectly categorised one patient as infected. Notably, it was on the absence of a positive microbiology culture. This patient, having been followed up for more than five years, has not developed any clinical signs of infection. On review, some of the neutrophils identified by the computer model were considered to represent necrotic fragmented cells rather than neutrophils (false positive).

Confusion tables comparing the model’s identifications with histological, microbiological, and ultimate MDT diagnosis showed no significant difference in classifications (p-values 1, 1, and 1, respectively; McNemar test). However, this must be interpreted with caution as the precision, recall, and F1 score give a better impression of the model’s performance.

## Discussion

This study has tested the ability of the YOLO 11x model’s capacity to identify neutrophils within digital images of H&E-stained FFPE pathological slides from samples taken at the time of revision arthroplasty. The trained network performed well in identifying neutrophils within the histology slides upon which they were assessed. When evaluated, it demonstrated comparable precision and recall to conventional histology, microbiological evaluation, and MDT opinion.

Compared to traditional methods involving the pathologist identifying and counting neutrophils, this method has the benefit of identifying neutrophils in each high-powered field across the whole slide image instead of a recommended sample of five or ten high-powered areas. Given the variability of neutrophils within the tissue, this gives this method an advantage of showing the distribution of neutrophils within the tissue and directing pathologists to neutrophil ‘hot spots’.

Identification of neutrophils can be challenging, and opinion is known to vary between pathologists. It is therefore interesting that the trained model demonstrated a better precision, recall, and F1 score on the patch than the interobserver variation between two of the authors assessing the same image.

Using neutrophil identifications as a marker of infection against the ultimate clinical diagnosis, it is notable that out of the 19 cases there was one false positive and two false negatives. Recall, precision, and F1 score are within the bounds of what is achieved by means of other methods for diagnosis of PJI (85% to 92%).^56^

The results of this study align the trained YOLO 11x model with other models assessed in studies evaluating machine-learning methods to assess pathological sections. This mirrors work by Alam and Islam^37^ in its demonstration of tinyYOLO model giving excellent results in identification and classification of blood cells within a blood film, with the efficientNet V2-S and CAMEL2 models in PJI,^47^ and with ResNet50 in evaluation of a blood smear.^38,40^

When our model is compared to the models used by Tao et al,^47^ there are similarities and differences. The study employed differing methods for training a range of models. Their best-performing model had an impressive sensitivity of 96% and a specificity of 91%. In their supervised models, they described areas with features of infection being marked with boundary boxes, and the returned information as a heat map to diagnose infection. This differs from our model, which is trained with the specific objective of identifying each neutrophil within the sample and returning a numerical value for neutrophil count which must be interpreted. The final decision regarding the presence of absence of infection is made by a human observer rather than an algorithm.

Our model can draw a pathologist’s attention to an area within a section suspected of having high numbers of neutrophils and other signs of infection (such as the presence of plasma cells and lymphocytes),^19,57^ employing the same principles used in Path AI, an FDA-approved tool, for examining prostate biopsy specimens.^44,45,58^ Corrective annotations can then be fed into a future training set to continue to improve the model.

The threshold for identification of a neutrophil polymorph by the trained model was set at 50% confidence. This has demonstrated good sensitivity. Increasing this threshold may reduce false positives, and may be possible with continued training of the model and exposure to further cases.

Potential applications for this model could include automating cell counts within the specimens prior to formal evaluation of a pre-marked and labelled image by the pathologist, or used as an independent marker for aiding the diagnosis of infection. An advantage of using YOLO is that it is fast enough for real-time detection, and therefore could be mounted onto the optics of a microscope, allowing for areas of high neutrophil density to be labelled and attention drawn to a suspicious area.

In conventional light microscopy, the pathologist focuses up and down to help with the identification of neutrophils (due to slide thickness). However, this is not possible in digital images, as the focus is fixed.

Images from the downloaded datasets had a 100× magnification and a relative uniform background. However, histology slides are scanned at 40× magnification. The number of pixels occupied by a neutrophil were different. Consequently, images were scaled so that a neutrophil occupied approximately 40 pixels. Although downscaling has the concern of losing information, in practice this worked better than upscaling.

Following completion of training, YOLO 11x demonstrated effective generalization to identification of neutrophils in the histology slides.

All neutrophils in a slide are counted, including neutrophils that may be present in blood vessels or granulation tissue. Consequently, it is important to keep the pathologist in the loop. It is anticipated that the model will be improved with further feedback and retraining of misclassified images.

In this study, sections from all tested patients were stained in the same laboratory and scanned on the same machine. Procedures and equipment vary between laboratories, thus before being able to apply the results to a wider population further prospective evaluation, using different laboratories and scanners as well as within different populations, is required to validate the model. Further training on discrepancies is likely to be needed as they arise, to facilitate continued improvement of the model’s performance. Additionally, it would be valuable to include frozen sections in model training and generalisation across different tissue processing techniques. Naphthol AS-D-chloroacetate enzyme staining could help to identify neutrophils as the ground truth in frozen sections.

Histological specimens all came from cases where samples were taken as part of the workup for infection or taken at the time of revision surgery. Not all cases of revision arthroplasty will have samples taken. This, therefore, has the potential to introduce an element of selection bias.

The images of neutrophils within the three training datasets were all of circulating neutrophils, and therefore are morphologically similar but marginally smaller in size. Despite this difference, the model performed well in identification of neutrophils.

The next step to move this project forward would be to undertake a multicentre study involving a range of revision units, laboratories, and scanners to further test the model’s generalizability and use in different settings.

The model presented in this study was trained on publicly available blood image datasets which were augmented with annotated histology images patches; it demonstrates that neutrophils can be accurately counted in scanned whole histology slides, aiding the diagnosis of PJI. However, keeping the pathologist in the loop is necessary to maintain oversight as well as to improve model performance by updating the training set with annotated misclassifications. Furthermore, clinical validation using different laboratories, tissue processing techniques, and scanners is required. The trained model provides an interesting area for further research which, with further development and validation, could be introduced into the workflow of pathology departments. Its practical use has yet to be defined.

## Data Availability

The data for this study are publicly available at

## References

[1] Vrancianu CO Serban B Gheorghe-Barbu I et al. The challenge of periprosthetic joint infection diagnosis: from current methods to emerging biomarkers Int J Mol Sci 2023 24 5 4320 10.3390/ijms24054320 36901750 PMC10002145

[2] Pellegrini A Legnani C Meani E A new perspective on current prosthetic joint infection classifications: introducing topography as a key factor affecting treatment strategy Arch Orthop Trauma Surg 2019 139 3 317 322 10.1007/s00402-018-3058-y 30374532 PMC6394468

[3] Cai Y Liang J Chen X et al. Synovial fluid neutrophil extracellular traps could improve the diagnosis of periprosthetic joint infection Bone Joint Res 2023 12 2 113 120 10.1302/2046-3758.122.BJR-2022-0391.R1 36718647 PMC9950667

[4] Jandl NM Kleiss S Mussawy H Beil FT Hubert J Rolvien T Absolute synovial polymorphonuclear neutrophil cell count as a biomarker of periprosthetic joint infection Bone Joint J 2023 105-B 4 373 381 10.1302/0301-620X.105B4.BJJ-2022-0628.R1 36924172

[5] Wang Y Li G Ji B et al. Diagnosis of periprosthetic joint infections in patients who have rheumatoid arthritis Bone Joint Res 2023 12 9 559 570 10.1302/2046-3758.129.BJR-2022-0432.R1 37704202 PMC10499527

[6] Denyer S Eikani C Sheth M Schmitt D Brown N Diagnosing periprosthetic joint infection Bone Jt Open 2023 4 11 881 888 10.1302/2633-1462.411.BJO-2023-0094.R1 37984446 PMC10659814

[7] Oto J Herranz R Fuertes M et al. Dysregulated neutrophil extracellular traps and haemostatic biomarkers as diagnostic tools and therapeutic targets in periprosthetic joint infection Bone Joint J 2024 106-B 9 1021 1030 10.1302/0301-620X.106B9.BJJ-2024-0187.R1 39216868

[8] Parvizi J Zmistowski B Berbari EF et al. New definition for periprosthetic joint infection: from the Workgroup of the Musculoskeletal Infection Society Clin Orthop Relat Res 2011 469 11 2992 2994 10.1007/s11999-011-2102-9 21938532 PMC3183178

[9] Schwarz EM Parvizi J Gehrke T et al. 2018 International Consensus Meeting on musculoskeletal infection: research priorities from the general assembly questions J Orthop Res 2019 37 5 997 1006 10.1002/jor.24293 30977537

[10] Parvizi J Tan TL Goswami K et al. The 2018 definition of periprosthetic hip and knee infection: an evidence-based and validated criteria J Arthroplasty 2018 33 5 1309 1314 10.1016/j.arth.2018.02.078 29551303

[11] McNally M Sousa R Wouthuyzen-Bakker M et al. The EBJIS definition of periprosthetic joint infection: a practical guide for clinicians Bone Joint J 2021 103-B 1 18 25 10.1302/0301-620X.103B1.BJJ-2020-1381.R1 33380199 PMC7954183

[12] Sigmund IK McNally MA Luger M Böhler C Windhager R Sulzbacher I Diagnostic accuracy of neutrophil counts in histopathological tissue analysis in periprosthetic joint infection using the ICM, IDSA, and EBJIS criteria Bone Joint Res 2021 10 8 536 547 10.1302/2046-3758.108.BJR-2021-0058.R1 34409845 PMC8414440

[13] Musso AD Mohanty K Spencer-Jones R Role of frozen section histology in diagnosis of infection during revision arthroplasty Postgrad Med J 2003 79 936 590 593 10.1136/pmj.79.936.590 14612604 PMC1742852

[14] Spangehl MJ Masri BA O’Connell JX Duncan CP Prospective analysis of preoperative and intraoperative investigations for the diagnosis of infection at the sites of two hundred and two revision total hip arthroplasties J Bone Joint Surg Am 1999 81-A 5 672 683 10.2106/00004623-199905000-00008 10360695

[15] Bori G McNally MA Athanasou N Histopathology in periprosthetic joint infection: when will the morphomolecular diagnosis be a reality? Biomed Res Int 2018 2018 1412701 10.1155/2018/1412701 29862251 PMC5971260

[16] Tigner A Ibrahim SA Murray IV Histology, White Blood Cell StatPearl 2022 https://www.ncbi.nlm.nih.gov/books/NBK563148/ date last accessed 13 November 2023

[17] Kashima TG Inagaki Y Grammatopoulos G Athanasou NA Use of chloroacetate esterase staining for the histological diagnosis of prosthetic joint infection Virchows Arch 2015 466 5 595 601 10.1007/s00428-015-1722-y 25687172

[18] Atiakshin D Kostin A Buchwalow I Morozow D Samoilova V Tiemann M Chloroacetate esterase reaction combined with immunohistochemical characterization of the specific tissue microenvironment of mast cells Histochem Cell Biol 2023 159 4 353 361 10.1007/s00418-022-02174-1 36598563 PMC10081974

[19] Uchihara Y Inagaki Y Munemoto M Tanaka Y Athanasou N Histological findings in infected and noninfected second stage revision knee arthroplasties J Knee Surg 2019 32 10 1015 1019 10.1055/s-0038-1675341 30396205

[20] McNally M Sousa R Wouthuyzen-Bakker M et al. The EBJIS definition of periprosthetic joint infection Bone Joint J 2021 103-B 1 18 25 10.1302/0301-620X.103B1.BJJ-2020-1381.R1 33380199 PMC7954183

[21] Feldman DS Lonner JH Desai P Zuckerman JD The role of intraoperative frozen sections in revision total joint arthroplasty J Bone Joint Surg Am 1995 77-A 12 1807 1813 10.2106/00004623-199512000-00003 8550647

[22] Athanasou NA Pandey R Steiger R McLardy Smith P The role of intraoperative frozen sections in revision total joint arthroplasty J Bone Joint Surg Am 1997 79-A 1433 1434 10.2106/00004623-199709000-00021 9314407

[23] Buttaro MA Martorell G Quinteros M Comba F Zanotti G Piccaluga F Intraoperative synovial C-reactive protein is as useful as frozen section to detect periprosthetic hip infection Clin Orthop Relat Res 2015 473 12 3876 3881 10.1007/s11999-015-4340-8 26013149 PMC4626517

[24] Grosso MJ Frangiamore SJ Ricchetti ET Bauer TW Iannotti JP Sensitivity of frozen section histology for identifying Propionibacterium acnes infections in revision shoulder arthroplasty J Bone Joint Surg Am 2014 96-A 6 442 447 10.2106/JBJS.M.00258 24647499

[25] Stroh DA Johnson AJ Naziri Q Mont MA Discrepancies between frozen and paraffin tissue sections have little effect on outcome of staged total knee arthroplasty revision for infection J Bone Joint Surg Am 2012 94-A 18 1662 1667 10.2106/JBJS.K.01600 22992877

[26] Morawietz L Tiddens O Mueller M et al. Twenty‐three neutrophil granulocytes in 10 high‐power fields is the best histopathological threshold to differentiate between aseptic and septic endoprosthesis loosening Histopathology 2009 54 7 847 853 10.1111/j.1365-2559.2009.03313.x 19635104

[27] Savarino L Tigani D Baldini N Bochicchio V Giunti A Pre-operative diagnosis of infection in total knee arthroplasty: an algorithm Knee Surg Sports Traumatol Arthrosc 2009 17 6 667 675 10.1007/s00167-009-0759-3 19259644

[28] Jiang H Ma H Qian W Gao M Li Y An automatic detection system of lung nodule based on multigroup patch-based deep learning network IEEE J Biomed Health Inform 2018 22 4 1227 1237 10.1109/JBHI.2017.2725903 28715341

[29] Havaei M Davy A Warde-Farley D et al. Brain tumor segmentation with deep neural networks Med Image Anal 2017 35 18 31 10.1016/j.media.2016.05.004 27310171

[30] Rajaraman S Kim I Antani SK Detection and visualization of abnormality in chest radiographs using modality-specific convolutional neural network ensembles PeerJ 2020 8 e8693 10.7717/peerj.8693 32211231 PMC7083159

[31] Winters J von Braunmuhl ME Zeemering S et al. JavaCyte, a novel open-source tool for automated quantification of key hallmarks of cardiac structural remodeling Sci Rep 2020 10 1 20074 10.1038/s41598-020-76932-3 33208780 PMC7675975

[32] Das A Nair MS Peter SD Sparse representation over learned dictionaries on the riemannian manifold for automated grading of nuclear pleomorphism in breast cancer IEEE Trans Image Process 2019 28 3 1248 1260 10.1109/TIP.2018.2877337 30346284

[33] Seeraj M Joy J A machine learning based framework for assisting pathologists in grading and counting of breast cancer cells ICT Express 2021 7 4 440 444 10.1016/j.icte.2021.02.005

[34] Vandenberghe ME Scott MLJ Scorer PW Söderberg M Balcerzak D Barker C Relevance of deep learning to facilitate the diagnosis of HER2 status in breast cancer Sci Rep 2017 7 1 45938 10.1038/srep45938 28378829 PMC5380996

[35] Khan AM Sirinukunwattana K Rajpoot N A global covariance descriptor for nuclear atypia scoring in breast histopathology images IEEE J Biomed Health Inform 2015 19 5 1637 1647 10.1109/JBHI.2015.2447008 26099150

[36] Korbar B Olofson AM Miraflor AP et al. Deep learning for classification of colorectal polyps on whole-slide images J Pathol Inform 2017 8 1 30 10.4103/jpi.jpi_34_17 28828201 PMC5545773

[37] Alam MM Islam MT Machine learning approach of automatic identification and counting of blood cells Healthc Technol Lett 2019 6 4 103 108 10.1049/htl.2018.5098 31531224 PMC6718065

[38] Chen YM Tsai JT Ho WH Automatic identifying and counting blood cells in smear images by using single shot detector and Taguchi method BMC Bioinformatics 2021 22 5 1 18 10.1186/s12859-022-05074-2 36482316 PMC9732976

[39] Lu C Ji M Ma Z Mandal M Automated image analysis of nuclear atypia in high-power field histopathological image J Microsc 2015 258 3 233 240 10.1111/jmi.12237 25787307

[40] Zhu Z Ren Z Lu S Wang S Zhang Y DLBCNet: a deep learning network for classifying blood cells Big Data Cogn Comput 2023 7 2 75 10.3390/bdcc7020075 38560757 PMC7615784

[41] Lin H Chen H Graham S Dou Q Rajpoot N Heng P-A Fast ScanNet: fast and dense analysis of multi-gigapixel whole-slide images for cancer metastasis detection IEEE Trans Med Imaging 2019 38 8 1948 1958 10.1109/TMI.2019.2891305 30624213

[42] Tingting Z Chen W Li S et al. Learning how to detect: a deep reinforcement learning method for whole-slide melanoma histopathology images Comput Med Imaging Graph 2023 108 102275 10.1016/j.compmedimag.2023.102275 37567046

[43] Redmon J Divvala S Girshick R Farhadi A You Only Look Once: Unified, Real-Time Object Detection 2016 IEEE Conference on Computer Vision and Pattern Recognition (CVPR) 2016 Las Vegas, Nevada, USA.

[44] Eloy C Marques A Pinto J et al. Artificial intelligence–assisted cancer diagnosis improves the efficiency of pathologists in prostatic biopsies Virchows Arch 2023 482 3 595 604 10.1007/s00428-023-03518-5 36809483 PMC10033575

[45] NICE Paige Prostate for prostate cancer Medtech innovation briefing 2021 www.nice.org.uk/guidance/mib280 date last accessed 31 January 2026

[46] Chen W Hu X Gu C et al. A machine learning-based model for “In-time” prediction of periprosthetic joint infection Digit Health 2024 10 20552076241253531 10.1177/20552076241253531 38766360 PMC11100394

[47] Tao Y Luo Y Hu H et al. Clinically applicable optimized periprosthetic joint infection diagnosis via AI based pathology NPJ Digit Med 2024 7 1 1 12 10.1038/s41746-024-01301-7 39462052 PMC11513062

[48] Tao Y Hu H Li J et al. A preliminary study on the application of deep learning methods based on convolutional network to the pathological diagnosis of PJI Arthroplasty 2022 4 1 49 10.1186/s42836-022-00145-4 36229852 PMC9563129

[49] No authors listed Human Tissue Act 2004 Legislation.gov.uk https://www.legislation.gov.uk/ukpga/2004/30/contents date last accessed 9 March 2026 10.12968/bjon.2006.15.15.21684

[50] World Medical Association World Medical Association Declaration of Helsinki: ethical principles for medical research involving human subjects JAMA 2013 310 20 2191 2194 10.1001/jama.2013.281053 24141714

[51] Mooney P Blood Cell Images 2017 https://www.kaggle.com/datasets/paultimothymooney/blood-cells date last accessed 28 January 2026

[52] Acevedo A Merino A Alférez S Molina Á Boldú L Rodellar J A dataset of microscopic peripheral blood cell images for development of automatic recognition systems Data Brief 2020 30 105474 10.1016/j.dib.2020.105474 32346559 PMC7182702

[53] Raabin Database Free data https://www.raabindata.com/free-data/ date last accessed 28 January 2026

[54] No authors listed GitHub - HumanSignal/labelImg https://github.com/HumanSignal/labelImg date last accessed 28 January 2026

[55] Kim D Pantanowitz L Schüffler P et al. (Re) defining the high-power field for digital pathology J Pathol Inform 2020 11 1 33 10.4103/jpi.jpi_48_20 33343994 PMC7737490

[56] Sigmund IK Luger M Windhager R McNally MA Diagnosing periprosthetic joint infections Bone Joint Res 2022 11 9 608 618 10.1302/2046-3758.119.BJR-2022-0078.R1 36047011 PMC9533249

[57] Bori G Muñoz-Mahamud E Garcia S et al. Interface membrane is the best sample for histological study to diagnose prosthetic joint infection Mod Pathol 2011 24 4 579 584 10.1038/modpathol.2010.219 21131917

[58] No authors listed 510(k) permanent notification U.S. Food & Drug Administration https://www.accessdata.fda.gov/scripts/cdrh/cfdocs/cfpmn/pmn.cfm?ID=K243391 date last accessed 31 January 2026

